# Lateral Sclerosis of the Spinal Cord

**Published:** 1884-12

**Authors:** J. E. Shaw

**Affiliations:** Physician to the Bristol Royal Infirmary


### LATERAL SCLEROSIS OF THE SPINAL CORD.

By J. E. Shaw, M.B., Physician to the Bristol Royal Infirmary.

A. B., æt. 13, admitted to the Bristol Infirmary, Feb. 7th, 1884.

*History.*—Patient is the fifth of nine children, of whom seven are still alive. Her mother states that when born the patient was a very large child, and that the labour at her birth was painful and tedious, lasting three days; the midwife in attendance then sent for a doctor, but before his arrival the child was born, so that no instrumental interference was resorted to. No special weakness or rigidity was noticed during infancy; but at two years of age, as she could not walk like other children, she was taken to Dr. Budd, of Worcester, who gave it as his opinion that it was due to laziness, and advised that the child should be encouraged to crawl about. When four years old she was taken to the Worcester Infirmary, attending there as an out-patient under Dr. English for two years, off and on; she was quite unable to walk, and was carried every day to and from school. At seven years of age she could read a little, and was not markedly behind other children in mental power. About six years ago her mother took her to the Birmingham Hospital for Children, where she was examined by Dr. Carter; admission as an in-patient was refused, but the mother was warned against letting the child be frightened or fall into the fire, and so forth. Shortly after this crutches were made for her, and she succeeded, for the first time, in walking without the assistance of another person. At that time she walked upon her toes, the heels being raised from the ground, as they are now. She has made no improvement in walking since then, but has progressed in intellectual development.

She has never had any kind of fit or convulsion. Has always been awkward in the use of her hands, especially of her right hand, and is therefore preponderatingly left-handed.

*State on Admission.*—Patient is somewhat below the average stature of her age, but not below the average of bulk and general development. Her eyes are staring, and her face expressionless, whence her appearance is rather that of an imbecile; this weakness of mind is, however, more apparent than real, as she is intelligent when led into conversation, and is a good-tempered and docile child.

When lying quietly in bed her legs are in a state of rigid extension, and strongly adducted, so that they cross one another if not kept forcibly apart. In sitting they can only be flexed to an obtuse angle, so that she sits with her legs stretched out, with the feet extended and the toes inverted. On being helped on to her feet, spasm of the muscles of the calf at once raises her on to her toes, the heels being elevated quite four inches from the ground. If she remains quiet this spasm gradually subsides, and the heels come lightly to the ground in something less than a minute. In attempting to walk, this upward jerk occurs at each step, causing a curious hopping gait; but at each step only one act of elevation occurs, not several alternating elevations and depressions. In moving forward, the toes cannot be completely raised from off the floor, but are scraped along it, and the spasm of the adductors of the thigh compels the passive leg and foot to be circumducted, as it were, round the other one by means of the abductors of the thigh, in order that it may be carried beyond the active one, across the front of which it is finally drawn. Patient cannot walk without some assistance; as she stoops forward so much, the support either of another person or of crutches is necessary to prevent her falling prone. No cutaneous plantar reflex is obtainable, and no contraction is produced by irritating the skin on the inner side of the thigh. No ankle clonus obtainable, apparently because the degree of tension in the tendo achillis is too great to permit of its occurrence. Patellar tendon reflex is exaggerated greatly beyond normal: the slightest tap upon the tendon, the body of the quadriceps extensor near the patella, or upon the upper part of the tibia, produces with curious suddenness one strong contraction of the quadriceps extensor, followed by two or three smaller ones; and a rapid succession of taps upon the patellar tendon keeps the limb extended in tonic rigidity for a short time. These phenomena are more marked in the right leg than in the left, which is rather less rigid than the right lower extremity. The muscles of the lower limbs are well developed, and show no sign of atrophy. Faradic current increases the rigidity without producing any distinct movement of the limbs; 10 cells of a constant-current battery give CCC very powerful, COC weak, ACC medium, and AOC strong. Sensation is quite unimpaired. Her hands and fore-arms are affected in a somewhat similar manner to her inferior extremities, though to a much less degree. In the hands and fingers there is little rigidity, but much loss of power. The fingers are capable of independent flexion and movement in the left hand, and to some degree so in the right hand also, but they are incapable of executing any performance more delicate than knitting, any attempt at sewing being a ludicrous blunder. The tendon and periosteal reflexes associated with the biceps, triceps, flexors and extensors of the fore-arms, and interossei are all greater than normal. Sensibility of the skin of fingers does not seem to be diminished, nor is there any marked tendency of the fingers to be clenched upon the palms, nor to the production of the *main en griffe.* The trunk of the body appears normal. There is no deep furrow separating the thorax and abdomen, and the abdominal skin reflexes are fairly well marked. The functions of the rectum and bladder are normal, and sexually she is developed somewhat precociously.

The speech is rather indistinct, from imperfect articulation, but there is no aphasia proper.

The girl remained under observation for some little time, but did not vary at all in her condition, and indeed does not seem to have done so of recent years, being in the stage in which most cases remain for a long time.

This case, so completely typical of its kind, I have described under the title of Lateral Sclerosis, because we should expect to find sclerosis of the pyramidal tracts, if not of the whole lateral columns, as the anatomical basis of the lesion, and because the disease is most generally known by that name. On the other hand, I have not ventured to call it a case of Primary Lateral Sclerosis, because the circumstances of the case tend rather to make one believe the sclerosis to be secondary, and because, also, primary lateral sclerosis of the spinal cord seems to remain still a sort of pathological *ignis fatuus,* especially now that Dr. Dreschfeld's case has proved, upon farther investigation, to have been complicated with changes in the ganglion cells of the anterior cornua.

The history of a lingering, difficult parturition process, the absence of motor power in the lower limbs during infancy, and the development of rigidity in them during the earlier years of childhood, lead one to the belief that this condition depends primarily upon a lesion of the excitomotor centres on each side of the brain (though not to the same degree on each side), caused by pressure of the parietal bones upon the subjacent convolutions at the time of labour. These psycho-motor centres having been more or less destroyed in the course of months, secondary degeneration spreads from them downwards through the internal capsule into the pyramids, and thence into the pyramidal tracts in the cord; or, perhaps more truly, these cerebral centres and pyramidal tracts in the cord do not proceed to complete normal evolution, as growth proceeds, after injury at birth; a condition to which Heschl has given the name of Porencephaly.

The case, therefore, is a variety of bi-hemiplegia, with late rigidity, if this theory be adopted as the correct one. The only point which seems to tell against the theory of bi-lateral hemiplegia is that the inferior extremities are much more severely affected than the superior. Dr. Gowers suggests to me that the overlapping of the edges of the sagittal suture causes more pressure upon the leg-centres, which lie nearer to the longitudinal fissure, than upon the arm-centres, which lie lower down.

Until a case of this description has been the subject of careful *post mortem* examination, some obscurity will continue to rest upon the precise extent and distribution of the lesion.

